# Myocarditis: Diagnostic Modalities and Treatment Options

**DOI:** 10.7759/cureus.79949

**Published:** 2025-03-03

**Authors:** Dihan Thilakaratne, Roshan Bista, Mark Zenker, Rohan Kaza, Sasan Raissi, Timir Paul

**Affiliations:** 1 Department of Internal Medicine, Ascension Saint Thomas Rutherford, Murfreesboro, USA; 2 Department of Cardiology, University of Tennessee Health Science Center, Ascension Saint Thomas Hospital West, Nashville, USA; 3 Department of Internal Medicine, St. George University School of Medicine, True Blue, GRD

**Keywords:** cmri cardiac magnetic resonance imaging, diagnostic testing, echocardiogram (echo), endomyocardial biopsy, myocarditis, etiology

## Abstract

Myocarditis is an underdiagnosed condition that affects people of all ages. It can be asymptomatic or present with a variety of symptoms. The etiology of myocarditis is broad and can be infectious, autoimmune, or toxin-induced. The diagnosis of myocarditis can be challenging at times due to varied clinical features that sometimes overlap with other cardiac conditions. It is essential to have a high index of suspicion and use appropriate diagnostic methods for timely detection. In this review, we discuss establishing the diagnosis of acute myocarditis with initial workup to gold standard noninvasive cardiac magnetic resonance imaging methods and the use of invasive techniques such as endomyocardial biopsy. Furthermore, we discuss the treatment options, including novel approaches based on the severity of the symptoms and the specific etiologies of myocarditis.

## Introduction and background

Myocarditis is characterized by inflammation within the myocardium and is associated with the degeneration and necrosis of myocytes of non-ischemic origin [1]. Acute myocarditis can be asymptomatic or present with arrhythmia or congestive heart failure. Patients with myocarditis can present with nonspecific symptoms of viral prodrome (fever, cough, cold, myalgia, or malaise) and more cardio-specific symptoms like palpitation, dyspnea, or chest pain. In its severe form, myocarditis leads to cardiogenic shock, which can be life-threatening. Fulminant myocarditis is the most severe manifestation of acute myocarditis which, unlike other forms of myocarditis, can progress rapidly, often leading to acute heart failure, cardiogenic shock, and even death [2,3].

The etiology of myocarditis is broad. It can be caused by infectious agents (such as viruses, bacteria, and protozoa), autoimmune diseases (lupus, scleroderma, Sjogren's syndrome), and drug toxicity. Table 1 illustrates the common causes of myocarditis. Recent data indicated myocarditis was one of the early comorbidities associated with coronavirus disease 2019 (COVID-19) infection and related to COVID-19 vaccination [4,5]. In addition, a case series also reported myocarditis in patients infected with monkeypox [6]. In this review, we focus on discussing the currently available diagnostic modalities and treatment options for managing myocarditis.

**Table 1:** Common causes of myocarditis IL-2: interleukin-2; COVID-19: coronavirus disease 2019

Infectious	Immune-Mediated	Toxin-Induced
Bacterial: *Streptococcus, Staphylococcus, Pneumococcus, Mycobacterium, Pneumococcus, Salmonella, Mycoplasma pneumoniae, Salmonella, Meningococcus, Borrelia, Leptospira*	Drugs: Penicillin, colchicine, eurosemide, thiazide diuretics, sulfonamides, tetracyclines, phenytoin, cefaclor	Amphetamines, cocaine, anthracyclines, cyclophosphamide, lithium, IL-2 drugs, trastuzumab
Viral: Coxsackieviruses A & B, Echovirus, Parvovirus B19, Cytomegalovirus, Human Herpes Virus-6, Poliovirus, Mumps, Rubella, Dengue, Yellow Fever, Respiratory syncytial virus, Hepatitis C virus, Varicella zoster virus, Monkeypox	Tetanus toxoid, vaccines (e.g., COVID-19 vaccines), serum sickness	Scorpion stings, spider bites, bee and wasp stings
Fungal: *Aspergillus*, *Candida, Actinomyces, Histoplasmosis, Blastomyces, Nocardia, Sporothrix*	Allo-antigens with heart transplant rejection	Radiation and electric shocks, pheochromocytoma, heavy metals
Parasites: *Trypanosoma cruzi, Toxoplasma gondii, Trichinella spiralis, Echinococcus granulosus, Leishmania*	Autoantigens with infection-negative giant cell, infection-negative lymphocytic	
	Autoimmune and immune-oriented disorders: Lupus, rheumatoid arthritis, inflammatory bowel disease, scleroderma, myasthenia gravis, sarcoidosis, Kawasaki’s disease, thyrotoxicosis, rheumatic heart diseases (rheumatic fever)	

## Review

Diagnostic modalities 

**Figure 1:**
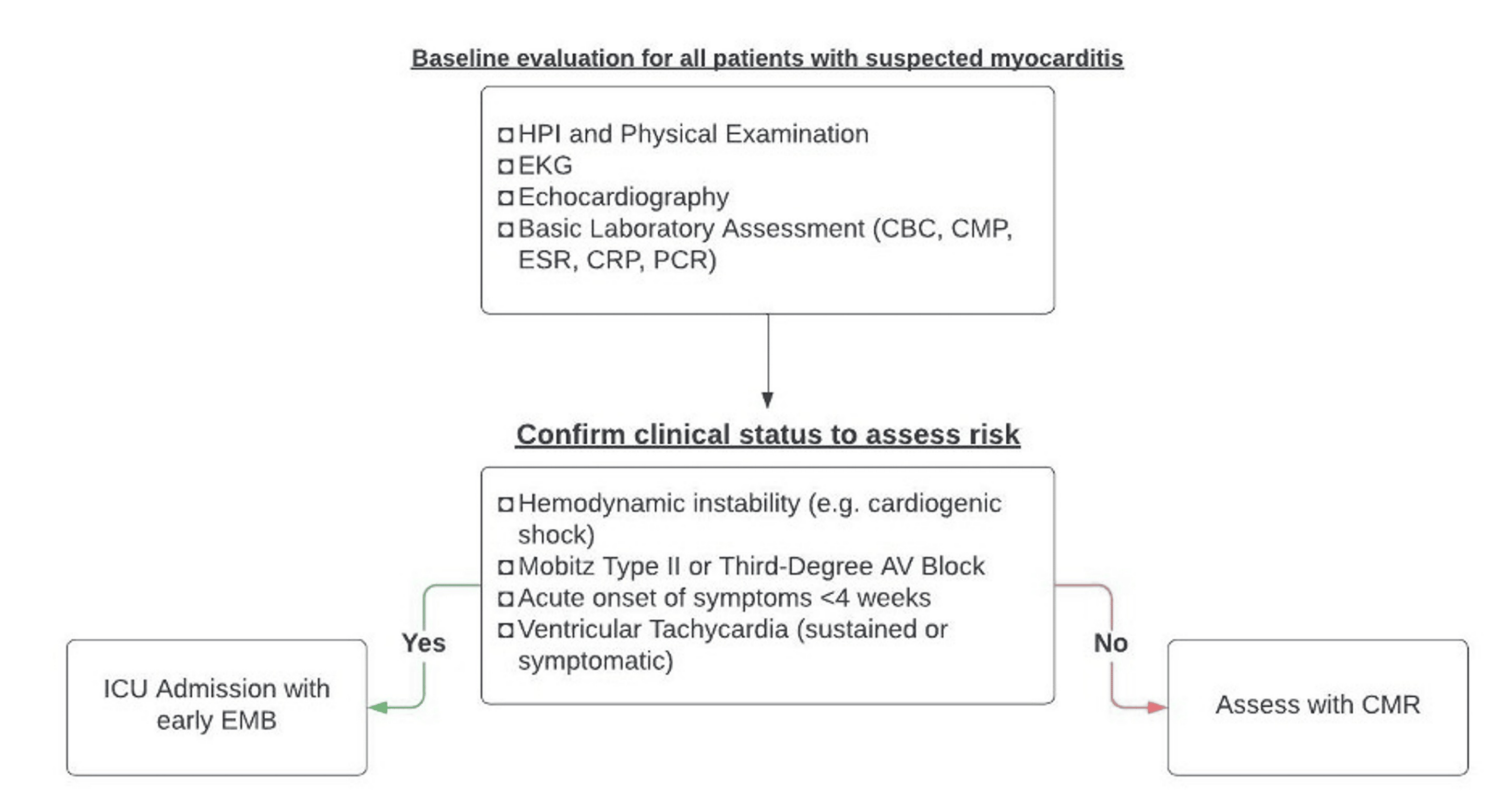
Diagnostic flowchart of myocarditis PCR: polymerase chain reaction; HPI: history of present illness; CBC: complete blood count; CMP: comprehensive metabolic panel; ESR: erythrocyte sedimentation rate; CRP: C-reactive protein; EMB: endomyocardial biopsy; CMR: cardiovascular magnetic resonance; ICU: intensive care unit

Establishing the diagnosis of acute myocarditis is challenging since there is no pathognomonic clinical presentation. When a patient is admitted with suspected myocarditis, a 12-lead electrocardiogram (ECG), echocardiogram (ECHO), complete blood count (CBC), comprehensive metabolic panel (CMP), brain natriuretic peptide (BNP), thyroid-stimulating hormone (TSH), troponin, inflammatory markers such as erythrocyte sedimentation rate (ESR) and C-reactive protein (CRP) in addition to extensive history, and physical exam should be obtained. Inflammatory markers such as ESR and CRP can often be elevated along with white counts. An autoimmune panel is needed if history and physical examination indicate autoimmune diseases. Viral serology is not routinely recommended in all patients but may be helpful in selected cases. However, it has no relevance in the diagnosis of myocarditis [7]. Troponin elevation in myocarditis is a marker of myocardial damage. In myocarditis, inflammatory mediators and immune cells infiltrate the myocardium causing myocardial necrosis and release of troponin in the blood stream [8]. Figure 1 shows a flow chart with the baseline evaluation for all patients with suspected myocarditis.

ECG Findings

The most common ECG abnormality in myocarditis is sinus tachycardia associated with nonspecific ST-T wave changes [9]. PR segment depression is a more frequent ECG finding in myopericarditis with pericarditis but is relatively less common in isolated myocarditis. High-degree atrioventricular (AV) block can be seen in fulminant myocarditis [10]. Frequent premature ventricular contractions may also suggest underlying myocardial inflammation related to myocarditis [11].

ECHO

Patients with clinically suspected myocarditis should undergo a transthoracic ECHO at initial presentation, which should be repeated during the hospitalization if there is any worsening of the patient's hemodynamic status [12]. Global ventricular systolic dysfunction and regional wall motion abnormalities may occur, but the left ventricular ejection fraction (LVEF) may be normal in a milder form [13]. According to the myocarditis treatment trial, active myocarditis is associated with left ventricular remodeling, such as dilation and reduced LVEF [14]. In contrast, fulminant myocarditis increases septal thickness [15]. Overall, ECHO findings of acute myocarditis can be nonspecific. However, given its noninvasive nature, comparing the data with other invasive and noninvasive modalities is helpful and necessary.

Cardiac Magnetic Resonance Imaging (CMRI) and Endomyocardial Biopsy (EMB)

Patients with suspected myocarditis often need further tests for the confirmation of diagnosis. This can be attained with CMRI or EMB. CMRI is currently being used in diagnosing and monitoring cardiovascular injury in patients with cancer, especially patients who have immune checkpoint-associated myocarditis [16]. Furthermore, CMRI is the current noninvasive gold standard for myocardial tissue characterization. It can detect image signal changes resulting from inflammation, including hyperemia, edema, capillary leak, necrosis, and fibrosis [17]. The inferolateral regions of the myocardium are known to be particularly susceptible to damage during myocarditis. The analysis of the kinetics of this area by CMRI provides better spatial resolution compared to the ECHO [18]. 

Based on these signal changes, with different myocardial characterizations, diagnostic criteria have been proposed as the Lake Louise Criteria [19]. Lake Louise Criteria were initially established in 2009 and revised in 2018 to standardize the diagnostic approach for acute myocarditis based on CMRI findings. It includes both major and supportive criteria for diagnosing acute myocarditis. CMRI indicates acute myocarditis if two out of three of Lake Louise Criteria are positive. Both vasodilation and cellular necrosis of myocyte connections can lead to vascular hyperpermeability in an inflamed myocardium. The affected myocardium rapidly absorbs contrast, which can be visualized by T1-weighted sequences on an MRI using early gadolinium enhancement (EGE) [20]. Intracellular edema allows for high water permeability in the myocardial tissue. T2 weighted sequence creates a high signal intensity in edematous tissue compared to the surrounding healthy muscle. 

CMRI showed the highest sensitivity among EMB-proven myocarditis cases in diagnosing myocarditis with infarct-like presentation (100%), while lower sensitivity in those with arrhythmias (50%) and heart failure (28%) [21]. Targets of regional wall abnormality and pericardial effusion with CMR steady state sequence are considered as "supportive only" under Lake Louise Criteria [22].

**Figure 2:**
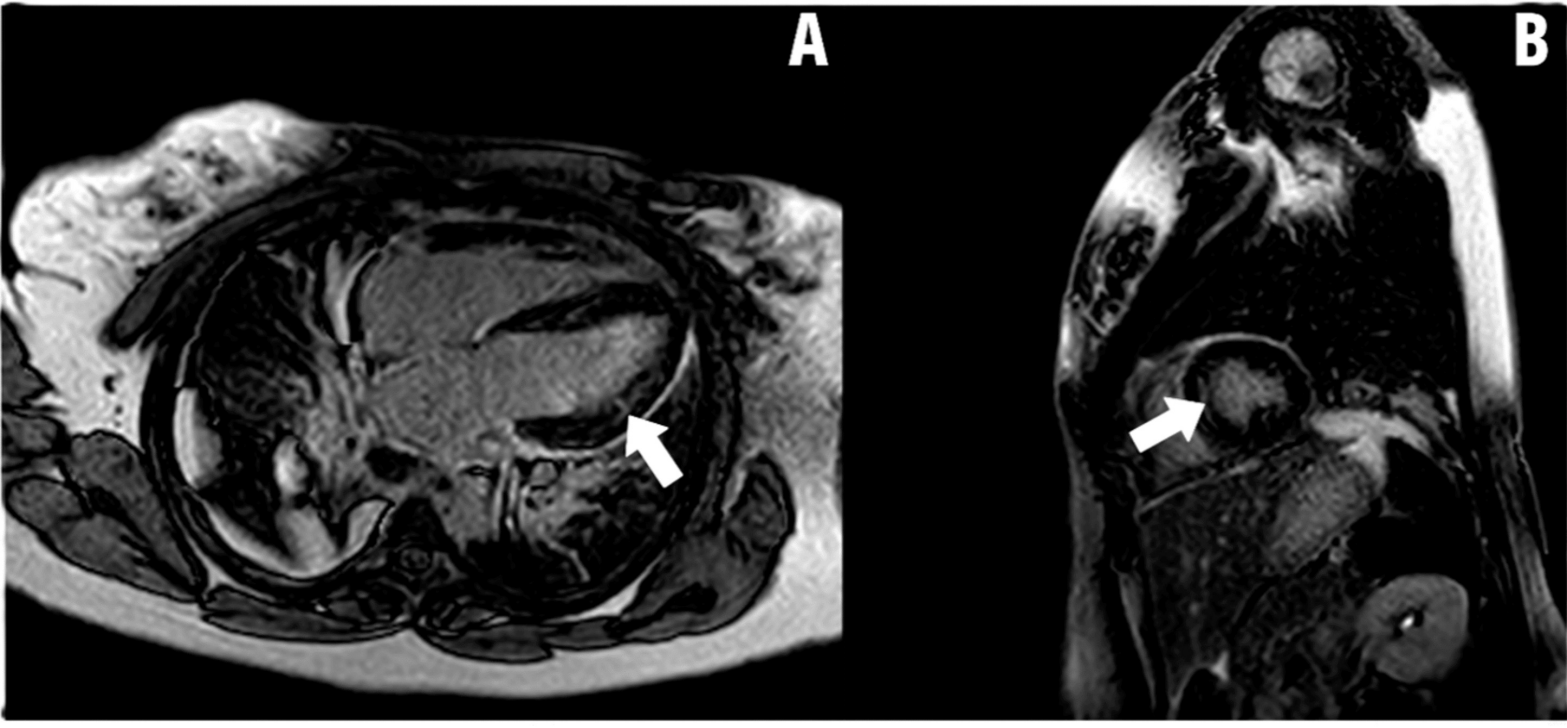
Cardiac MRI reveals both pericarditis and myocarditis. Both arrows point to heterogeneous mild myocardial delayed enhancement along with circumferential pericardial enhancement. Parametric mapping showed elevated myocardial native T1 time of 1154 ms and myocardial T2 time of 66 ms, consistent with acute perimyocarditis. (A) Horizontal long axis four-chamber anterolateral wall view; (B) Short axis view Image credit: Mark Zenker

**Figure 3:**
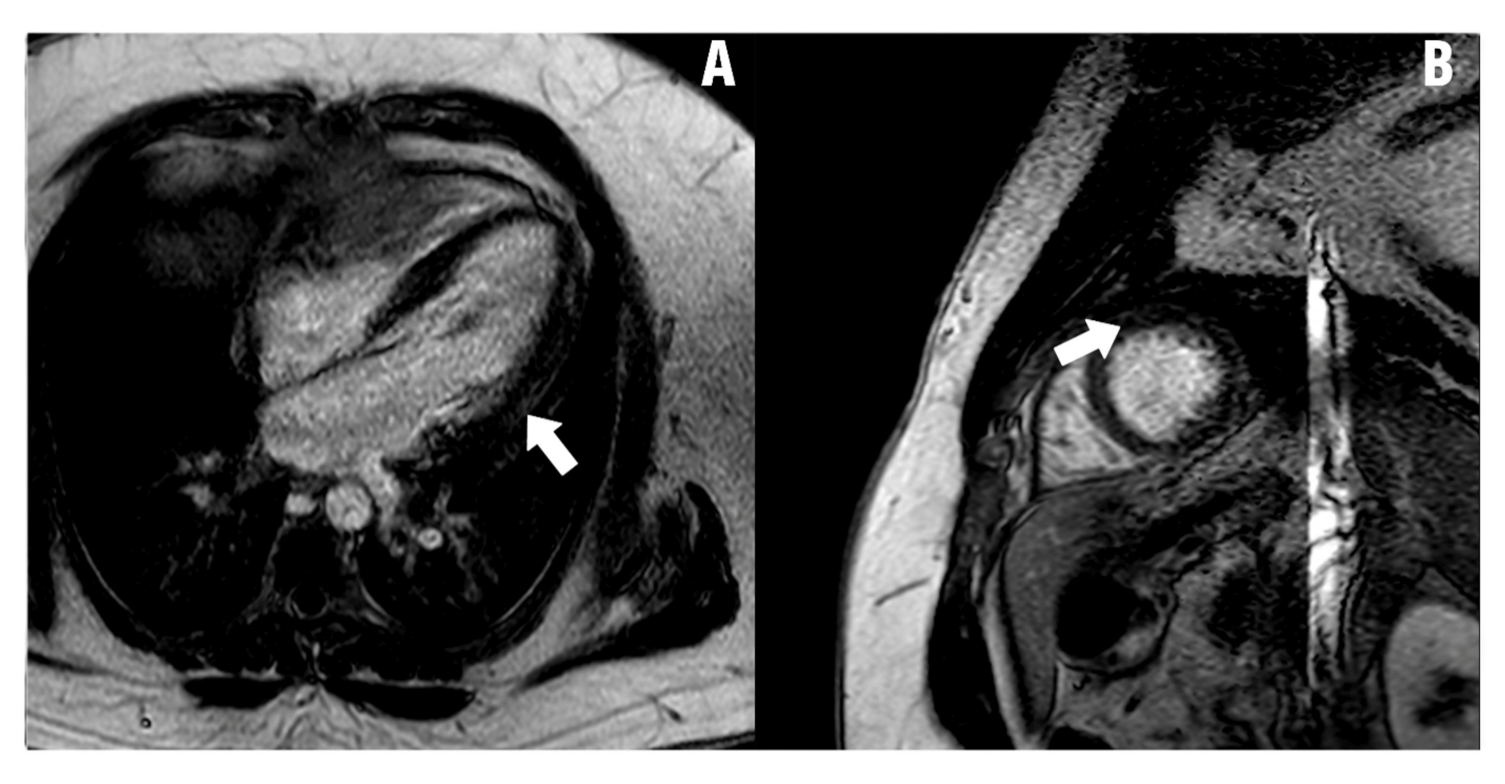
Cardiac MRI confirms acute myocarditis. Both arrows show acute myocarditis involving the left ventricle. According to the parametric mapping, myocardial native T1 time was 1022 ms and myocardial T2 time was 55 ms. (A) Horizontal long axis four chamber anterolateral wall view; (B) Short axis antero-apical wall view Image Credit: Mark Zenker

**Figure 4:**
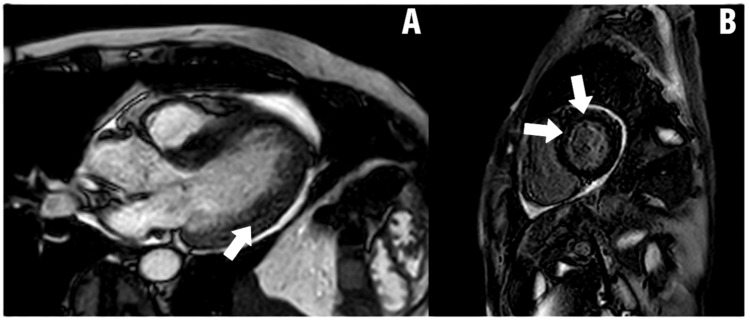
Cardiac MRI reveals acute peri-myocarditis. Arrows designate acute myocarditis involving the left ventricle. There is a diffuse mid myocardial delayed enhancement in all basal to apical segments along with pericardial enhancement. Moderate concentric left ventricular hypertrophy is also noted. According to the parametric mapping, there were significantly prolonged T2 and T1 mapping values. Myocardial native T1 time was 1250 ms and myocardial T2 time was 79 ms. (A) Horizontal long axis four chamber inferolateral wall view; (B) Short axis antero-apical and septal apical wall view Image Credit: Mark Zenker

Figures 2-4 show CMR images of clinically suspected myocarditis.

Positron Emission Tomography (PET)

PET with fluorodeoxyglucose (FDG) is a noninvasive and alternative diagnostic tool for patients contraindicated to CMRI. It is more beneficial in patients with myocarditis secondary to suspected systemic autoimmune diseases such as lupus and sarcoidosis [23,24].

EMB

EMB has an essential role in establishing the diagnosis of myocarditis. Improvements in EMB equipment and the development of new techniques for analyzing EMB samples have significantly improved diagnostic precision. EMB will be more informative when the pre-test likelihood is higher for suspected myocarditis and noninvasive tests are inconclusive. The American Heart Association published a statement recommending EMB as a first-line diagnostic modality for cases of any unexplained acute cardiomyopathy complicated by hemodynamic instability requiring mechanical ventilatory support, inotropic therapy, high-degree AV (atrioventricular) block, sustained ventricular tachycardia, or failure to respond to medical therapy within one to two weeks [25]. Standard diagnostic criteria, such as the Dallas criteria for myocarditis, have been used to provide the histopathological categorization and diagnosis of myocarditis for decades. It requires an inflammatory infiltrate and associated myocyte necrosis or damage not characteristic of an ischemic event [26]. Recent studies raise concern about whether the Dallas criteria are sensitive enough to identify a particular type of myocarditis. That does not mean complete exclusion of the histopathologic analysis and diagnosis of myocarditis. However, these findings do suggest that deeper immunotyping of myocardial inflammation may increase the diagnostic yield of the EMB.

Genetic Testing 

Clinicians support the consideration of genetic contributions to myocarditis. The inclusion of genetic testing for patients with cardiomyopathy, arrhythmias, family history of myocarditis, and sudden cardiac deaths identifies patients at a greater risk for recurrence of myocarditis and arrhythmia [27]. A retrospective study of myocardial-associated desmosome gene variants (DSV) showed adverse cardiovascular events as compared to patients without the variants [28]. Since acute myocarditis often mimics the clinical presentation of acute myocardial infarction, differentiating myocarditis from myocardial infarction can sometimes be challenging. With the recent identification of specific microRNA (miRNAs-endogenous single-stranded non-coding RNA) in mice and humans with myocarditis, novel data showed that human homolog (has-miR Ch8:96) could be used to distinguish patients with myocarditis from patients with myocardial infarction [29]. miRNAs are considered pivotal epigenetic regulators of heart function, influencing cardiac differentiation, proliferation, injury, and inflammation [30,31]. However, further prospective studies would be helpful to ascertain if genetic testing could improve the risk stratification of patients with myocarditis who are considered low-risk.

Treatment options

Myocarditis can present as mild-moderate acute heart failure to cardiogenic shock and ventricular arrhythmias. It can present as fulminant myocarditis, acute myocarditis, or chronic myocarditis. The treatment for myocarditis is based on the severity of symptoms and the specific etiologies of myocarditis. It can range from conservative management to the need for mechanical circulatory support and heart transplantation. 

General Measures

Intense physical activity should be avoided for three to six months after acute myocarditis diagnosis [32].

Immunosuppression

Empiric use of intravenous (IV) corticosteroids is an integral part of management in patients with fulminant myocarditis and acute myocarditis causing acute heart failure, high-degree atrioventricular block, or ventricular arrhythmias [33]. Maintenance immunosuppressive agents are often required for autoimmune conditions leading to myocarditis, such as sarcoidosis, giant cell myocarditis, or eosinophilic myocarditis.

Intravenous immunoglobulin (IVIG)

IVIG has immune-modulating, anti-infectious, and antioxidative stress effects [34]. Improving the clinical course may be due to the modulation of inflammatory cytokines and peripheral leukocytes [35]. 

Management of Acute Heart Failure

Myocarditis can lead to heart failure with reduced ejection fraction (HFrEF); patients with HFrEF should be treated with four pillars of guideline-directed medical therapy(GDMT) as tolerated, including angiotensin-converting enzyme inhibitors (ACEi), angiotensin receptor blockers (ARB), and angiotensin receptor neprilysin inhibitor (ARNi), magnetic resonance angiography (MRA), sodium-glucose cotransporter 2 inhibitor (SGLT2i), and beta blockers [36].

Management of Ventricular Arrhythmias and AV Block

Ventricular arrhythmia, high-degree AV block, and death are common with giant cell myocarditis, sarcoidosis, immune checkpoint-associated myocarditis, and eosinophilic myocarditis. Thus, the prompt diagnosis of these specific causes of myocarditis is essential for timely management [37,38]. Based on etiologies and presentations, these patients may require antiarrhythmics, implantable cardioverter defibrillator (ICD)/pacemaker, or ablation. Furthermore, the three-dimensional reconstruction of CMRI guides intra-procedural electroanatomic mapping before catheter ablation, which has gained an increasing role in identifying and treating scar areas [39].

Mechanical Circulatory Support and Heart Transplant

Patients refractory to medical treatment and with ongoing hemodynamic deterioration may require inotropic support and mechanical circulatory support as a bridging or destination therapy for heart transplant, especially for those with fulminant giant cell myocarditis [40].

Etiology-Specific Treatment for Myocarditis 

Infectious: Acute myocarditis can be caused by many infectious agents, including viral, protozoal, fungal, and bacterial, with viral myocarditis being the most common form of infectious myocarditis. Most cases of viral myocarditis are diagnosed after several weeks of viral infection when viral clearance is already achieved. So, anti-viral are rarely used. If reverse transcription polymerase chain reaction (RT-PCR) shows the persistence of viral infection, especially adenovirus or enterovirus, then interferon beta can improve ventricular function. Direct-acting anti-retroviral therapy can be initiated in patients with hepatitis C virus (HCV), human immunodeficiency virus (HIV), or influenza viruses [41]. Therapies for COVID-19-related myocarditis are mostly supportive treatments and those extrapolated from the standard of practice for non-COVID-19-related myocarditis [42].

Immune-mediated myocarditis: Giant cell myocarditis: Immunosuppressive medications include anti-thymocyte globulin, calcineurin inhibitors, and high-dose corticosteroids [43].

Eosinophilic myocarditis: Immunosuppression with corticosteroids alone or in combination with azathioprine or sole use of cyclophosphamide or methotrexate [44].

Myocarditis due to sarcoidosis: Steroids are first-line drugs [45]. Methotrexate or mycophenolate mofetil can be used as a second-line agent if the patient is refractory to steroids, but side effects limit its use. 

Immune-checkpoint inhibitor (ICI)-associated myocarditis: It's imperative to stop ICIs and start IV corticosteroids. Immunosuppressive agents that can be used are anti-cluster of differentiation-52 (anti-CD52) antibodies (alemtuzumab), anti-cluster of differentiation-3 (anti-CD3) antibodies (anti-thymocyte globulin), or cytotoxic T lymphocyte antigen-4 (CTLA-4) agonist (abatacept) [46].

Systemic autoimmune disorder-associated myocarditis: Corticosteroids in combination with IVIG, cyclophosphamide, or rituximab are effective during the acute phase. For maintenance therapy, mycophenolate mofetil, methotrexate, or azathioprine can be used [47].

## Conclusions

Myocarditis can range from mild viral forms to severe, life-threatening cases. Timely diagnosis is crucial for better outcomes, necessitating a high index of suspicion and the use of specific diagnostic tests. The initial workup typically includes a CBC, CMP, ECG, ECHO, and inflammatory markers such as ESR and CRP. Confirmatory tests may involve cardiac MRI and PET scans, which help identify abnormalities and autoimmune conditions, respectively. Treatment depends on the underlying cause and symptoms; many cases are self-limiting and require symptomatic care. Heart failure management includes guideline-directed medical therapy (GDMT), while severe cases may need mechanical support or cardiac transplantation. For immune-mediated myocarditis, intravenous glucocorticoids are often used, along with other immunosuppressive agents as needed. In cases associated with ICIs, stopping the therapy and administering high-dose corticosteroids is essential. Additional agents may be considered for ICI-associated myocarditis.

## References

[1] Montero S, Abrams D, Ammirati E (2022). Fulminant myocarditis in adults: a narrative review. J Geriatr Cardiol.

[2] Maisch B, Ruppert V, Pankuweit S (2014). Management of fulminant myocarditis: a diagnosis in search of its etiology but with therapeutic options. Curr Heart Fail Rep.

[3] Fairweather D, Beetler DJ, Di Florio DN, Musigk N, Heidecker B, Cooper LT Jr (2023). COVID-19, myocarditis and pericarditis. Circ Res.

[4] Castiello T, Georgiopoulos G, Finocchiaro G (2022). COVID-19 and myocarditis: a systematic review and overview of current challenges. Heart Fail Rev.

[5] Cho JY, Kim KH, Lee N (2023). COVID-19 vaccination-related myocarditis: a Korean nationwide study. Eur Heart J.

[6] Dumont M, Guilhou T, Gerin M (2023). Myocarditis in monkeypox-infected patients: a case series. Clin Microbiol Infect.

[7] Mahfoud F, Gärtner B, Kindermann M (2011). Virus serology in patients with suspected myocarditis: utility or futility?. Eur Heart J.

[8] Caforio AL, Pankuweit S, Arbustini E (2013). Current state of knowledge on aetiology, diagnosis, management, and therapy of myocarditis: a position statement of the European Society of Cardiology Working Group on Myocardial and Pericardial Diseases. Eur Heart J.

[9] Buttà C, Zappia L, Laterra G, Roberto M (2020). Diagnostic and prognostic role of electrocardiogram in acute myocarditis: a comprehensive review. Ann Noninvasive Electrocardiol.

[10] Qin Z, Luo L, Ge L (2023). Electrocardiogram of a patient with mushroom poisoning-induced myocarditis. Ann Noninvasive Electrocardiol.

[11] Lakkireddy D, Turagam MK, Yarlagadda B (2019). Myocarditis causing premature ventricular contractions: insights from the MAVERIC registry. Circ Arrhythm Electrophysiol.

[12] Tschöpe C, Cooper LT, Torre-Amione G, Van Linthout S (2019). Management of myocarditis-related cardiomyopathy in adults. Circ Res.

[13] Kostakou PM, Kostopoulos VS, Tryfou ES, Giannaris VD, Rodis IE, Olympios CD, Kouris NT (2018). Subclinical left ventricular dysfunction and correlation with regional strain analysis in myocarditis with normal ejection fraction. A new diagnostic criterion. Int J Cardiol.

[14] Mendes LA, Picard MH, Dec GW, Hartz VL, Palacios IF, Davidoff R (1999). Ventricular remodeling in active myocarditis. Myocarditis treatment trial. Am Heart J.

[15] Felker GM, Boehmer JP, Hruban RH, Hutchins GM, Kasper EK, Baughman KL, Hare JM (2000). Echocardiographic findings in fulminant and acute myocarditis. J Am Coll Cardiol.

[16] Cau R, Solinas C, De Silva P (2022). Role of cardiac MRI in the diagnosis of immune checkpoint inhibitor-associated myocarditis. Int J Cancer.

[17] Thomas KE, Fotaki A, Botnar RM, Ferreira VM (2023). Imaging methods: magnetic resonance imaging. Circ Cardiovasc Imaging.

[18] Nagel E, Kwong RY, Chandrashekhar YS (2020). CMR in nonischemic myocardial inflammation: solving the problem of diagnosing myocarditis or still diagnostic ambiguity?. JACC Cardiovasc Imaging.

[19] Ferreira VM, Schulz-Menger J, Holmvang G (2018). Cardiovascular magnetic resonance in nonischemic myocardial inflammation: expert recommendations. J Am Coll Cardiol.

[20] Daneshrad JA, Ordovas K, Sierra-Galan LM, Hays AG, Mamas MA, Bucciarelli-Ducci C, Parwani P (2023). Role of cardiac magnetic resonance imaging in the evaluation of MINOCA. J Clin Med.

[21] Baritussio A, Cheng CY, Simeti G (2024). CMR predictors of favorable outcome in myocarditis: a single-center experience. J Clin Med.

[22] Friedrich MG, Marcotte F (2013). Cardiac magnetic resonance assessment of myocarditis. Circ Cardiovasc Imaging.

[23] Chareonthaitawee P, Beanlands RS, Chen W (2017). Joint SNMMI-ASNC expert consensus document on the role of (18)F-FDG PET/CT in cardiac sarcoid detection and therapy monitoring. J Nucl Med.

[24] Seferović PM, Tsutsui H, McNamara DM (2021). Heart Failure Association of the ESC, Heart Failure Society of America and Japanese Heart Failure Society position statement on endomyocardial biopsy. Eur J Heart Fail.

[25] van der Boon RM, den Dekker WK, Meuwese CL (2021). Safety of endomyocardial biopsy in new-onset acute heart failure requiring veno-arterial extracorporeal membrane oxygenation. Circ Heart Fail.

[26] Baughman KL (2006). Diagnosis of myocarditis: death of Dallas criteria. Circulation.

[27] McNally EM, Selgrade DF (2022). Genetic testing for myocarditis. JACC Heart Fail.

[28] Ammirati E, Raimondi F, Piriou N (2022). Acute myocarditis associated with desmosomal gene variants. JACC Heart Fail.

[29] Blanco-Domínguez R, Sánchez-Díaz R, de la Fuente H (2021). A novel circulating microRNA for the detection of acute myocarditis. N Engl J Med.

[30] Tymińska A, Ozierański K, Skwarek A (2022). Personalized management of myocarditis and inflammatory cardiomyopathy in clinical practice. J Pers Med.

[31] Aleshcheva G, Pietsch H, Escher F, Schultheiss HP (2021). MicroRNA profiling as a novel diagnostic tool for identification of patients with inflammatory and/or virally induced cardiomyopathies. ESC Heart Fail.

[32] Pelliccia A, Solberg EE, Papadakis M (2019). Recommendations for participation in competitive and leisure time sport in athletes with cardiomyopathies, myocarditis, and pericarditis: position statement of the sport cardiology section of the European Association of Preventive Cardiology (EAPC). Eur Heart J.

[33] Ammirati E, Frigerio M, Adler ED (2020). Management of acute myocarditis and chronic inflammatory cardiomyopathy: an expert consensus document. Circ Heart Fail.

[34] Huang X, Sun Y, Su G, Li Y, Shuai X (2019). Intravenous immunoglobulin therapy for acute myocarditis in children and adults. Int Heart J.

[35] Kishimoto C, Shioji K, Hashimoto T (2014). Therapy with immunoglobulin in patients with acute myocarditis and cardiomyopathy: analysis of leukocyte balance. Heart Vessels.

[36] Heidenreich PA, Bozkurt B, Aguilar D (2022). 2022 AHA/ACC/HFSA guideline for the management of heart failure: a report of the American College of Cardiology/American Heart Association joint committee on clinical practice guidelines. Circulation.

[37] Peretto G, Sala S, Rizzo S (2019). Arrhythmias in myocarditis: state of the art. Heart Rhythm.

[38] Könemann H, Dagres N, Merino JL, Sticherling C, Zeppenfeld K, Tfelt-Hansen J, Eckardt L (2023). Spotlight on the 2022 ESC guideline management of ventricular arrhythmias and prevention of sudden cardiac death: 10 novel key aspects. Europace.

[39] Schwarzl JM, Schleberger R, Kahle AK (2021). Specific electrogram characteristics impact substrate ablation target area in patients with scar-related ventricular tachycardia-insights from automated ultrahigh-density mapping. J Cardiovasc Electrophysiol.

[40] Brailovsky Y, Masoumi A, Bijou R, Oliveros E, Sayer G, Takeda K, Uriel N (2022). Fulminant giant cell myocarditis requiring bridge with mechanical circulatory support to heart transplantation. JACC Case Rep.

[41] Baik SH, Jeong HS, Kim SJ, Yoon YK, Sohn JW, Kim MJ (2015). A case of influenza associated fulminant myocarditis successfully treated with intravenous peramivir. Infect Chemother.

[42] Lovell JP, Čiháková D, Gilotra NA (2022). COVID-19 and myocarditis: review of clinical presentations, pathogenesis and management. Heart Int.

[43] Naseeb MW, Adedara VO, Haseeb MT (2023). Immunomodulatory therapy for giant cell myocarditis: a narrative review. Cureus.

[44] Aggarwal A, Bergin P, Jessup P, Kaye D (2001). Hypersensitivity myocarditis presenting as cardiogenic shock. J Heart Lung Transplant.

[45] Gilotra NA, Griffin JM, Pavlovic N (2022). Sarcoidosis-related cardiomyopathy: current knowledge, challenges, and future perspectives state-of-the-art review. J Card Fail.

[46] Moslehi J, Lichtman AH, Sharpe AH, Galluzzi L, Kitsis RN (2021). Immune checkpoint inhibitor-associated myocarditis: manifestations and mechanisms. J Clin Invest.

[47] Tschöpe C, Ammirati E, Bozkurt B (2021). Myocarditis and inflammatory cardiomyopathy: current evidence and future directions. Nat Rev Cardiol.

